# IL-34 Actions on FOXP3^+^ Tregs and CD14^+^ Monocytes Control Human Graft Rejection

**DOI:** 10.3389/fimmu.2020.01496

**Published:** 2020-08-11

**Authors:** Séverine Bézie, Antoine Freuchet, Céline Sérazin, Apolline Salama, Nadège Vimond, Ignacio Anegon, Carole Guillonneau

**Affiliations:** ^1^Centre de Recherche en Transplantation et Immunologie UMR1064, INSERM, Université de Nantes, Nantes, France; ^2^Institut de Transplantation Urologie Néphrologie (ITUN), CHU Nantes, Nantes, France; ^3^LabEx IGO “Immunotherapy, Graft, Oncology”, Nantes, France

**Keywords:** IL-34, transplantation, tolerance, monocyte, Treg, cell therapy, GVHD, CSF-1R

## Abstract

Cytokines are major players regulating immune responses toward inflammatory and tolerogenic results. In organ and bone marrow transplantation, new reagents are needed to inhibit tissue destructive mechanisms and eventually induce immune tolerance without overall immunosuppression. IL-34 is a cytokine with no significant homology with any other cytokine but that acts preferentially through CSF-1R, as CSF-1 does, and through PTPζ and CD138. Although IL-34 and CSF-1 share actions, a detailed analysis of their effects on immune cells needs further research. We previously showed that both CD4^+^ and CD8^+^ FOXP3^+^ Tregs suppress effector T cells through the production of IL-34, but not CSF-1, and that this action was mediated through antigen-presenting cells. We showed here by single-cell RNAseq and cytofluorimetry that different subsets of human monocytes expressed different levels of CSF-1R, CD138, and PTPζ and that both CD4^+^ and CD8^+^ FOXP3^+^ Tregs expressed higher levels of CSF-1R than conventional T cells. The effects of IL-34 differed in the survival of these different subpopulations of monocytes and RNAseq analysis showed several genes differentially expressed between IL-34, CSF-1, M0, M1, and also M2 macrophages. Acute graft-vs.-host disease (aGVHD) in immunodeficient NSG mice injected with human PBMCs was decreased when treated with IL-34 in combination with an anti-CD45RC mAb that depleted conventional T cells. When IL-34-differentiated monocytes were used to expand Tregs *in vitro*, both CD4^+^ and CD8^+^ FOXP3^+^ Tregs were highly enriched and this effect was superior to the one obtained with CSF-1. Human CD8^+^ Tregs expanded *in vitro* with IL-34-differentiated allogeneic monocytes suppressed human immune responses in an NSG mouse aGVHD model humanized with hPBMCs. Overall, we showed that IL-34 induced the differentiation of human monocytes with a particular transcriptional profile and these cells favored the development of potent suppressor FOXP3^+^ Tregs.

## Introduction

Organ and bone marrow transplantation is the only treatment for patients suffering from a number of diseases. In organ transplantation, the use of immunosuppressors has allowed remarkable success in the short and medium term graft survival, but unwanted side effects still lead to high morbidity and mortality, even when avoiding excessive immunosuppression (1). In bone marrow transplantation, acute and chronic GVHD are very frequent complications with high mortality and morbidity and thus with high unmet clinical needs (2, 3). In the long term, immunosuppressors can even be deleterious in the establishment of tolerance (4). Therefore, new treatments are needed that will be more specific for allogeneic immune responses and/or induce fewer side effects and that would allow, at the least, to decrease the use of immunosuppressors. Cytokines and enzymes controlling metabolic pathways have been described as powerful tools for controlling immune responses and it is important to identify new mediators of immune tolerance. Interleukin-34 (IL-34) is a cytokine, described for the first time in 2008 (5). Although IL-34 shares no homology with macrophage colony-stimulating factor (CSF-1 or M-CSF) in its amino acid sequence, they share a common receptor (CSF-1R or CD115) and IL-34 also has two distinct receptors, protein-tyrosine-phosphatase zeta (PTPζ) and CD138 (syndecan-1) (6, 7), suggesting additional roles for IL-34. In addition, the affinity of IL-34 for CSF-1R is higher than the one of CSF-1 and the binding mode to CSF-1R, as well as signaling of both cytokines, are different (8). Until now, studies have demonstrated that IL-34 is released by some cell types and is involved in the differentiation and survival of macrophages, monocytes, and dendritic cells (DCs) in response to inflammation, in the development of microglia and Langerhans cells (9, 10). More recent articles have described the immunoregulatory properties of IL-34 (11, 12). We have demonstrated that IL-34 is secreted by FOXP3^+^ CD4^+^ and CD8^+^ regulatory T cells (Tregs) in human and CD8^+^CD45RC^low/−^ Tregs in rat. We also demonstrated that blockade of IL-34 *in vitro* in human and rat co-culture suppression assays inhibited both CD4^+^ and CD8^+^ Tregs suppressive function. Most importantly, we also showed that IL-34 treatment *in vivo* in a rat model of cardiac allograft induced transplant tolerance through the differentiation of macrophages toward a regulatory profile and subsequent induction of CD4^+^ and CD8^+^ Tregs by these macrophages (12). This role had never been evidenced before and needed to be explored in humans. We therefore investigated the tolerogenic effect of IL-34 on monocytes/macrophages and the mechanisms by which CD4^+^ and CD8^+^ Tregs were generated. Since CD4^+^ and CD8^+^ Tregs produce IL-34, our hypothesis was that IL-34 acts in autocrine and paracrine fashions to reinforce immune tolerance. Thus, we analyzed the expression of IL-34 receptors (CSF-1R, CD138, and PTPζ) on human monocytes and T cells and assessed the effect of IL-34 on human monocytes by single cell and bulk RNAseq. We also analyzed the effects of IL-34 on human Treg cell generation and evaluated in immune humanized mice the suppressive function of CD8^+^ Tregs differentiated using IL-34-treated human monocytes in a model of acute GVHD.

In the present manuscript we report that IL-34 can act on CD14^++^CSF-1R^+^PTPζ^+^ monocytes and CD4^+^ or CD8^+^ FOXP3^+^CSF-1R^+^ Tregs in an autocrine manner. We demonstrate that IL-34 action on monocytes results in differentiation toward a regulatory macrophage profile different from M2 macrophages, as shown by transcriptomic profiling. We demonstrate also that naive and effector precursor T cell depletion using anti-CD45RC mAbs results in synergistic enhanced IL-34 tolerogenic action *in vivo*. *In vitro*, we show that IL-34 is more efficient at inducing FOXP3^+^ Tregs than CSF-1 and that these FOXP3^+^ Tregs can efficiently control GVHD *in vivo* in a model of immune humanized immunodeficient mice.

Altogether, these data provide new informations on this new function of IL-34 on regulating Treg activity.

## Materials and Methods

### Healthy Volunteers' Blood Collection and PBMC Separation

Blood from healthy individuals was obtained at the Etablissement Français du Sang (Nantes, France). Written informed consent was provided according to institutional guidelines. Peripheral blood mononuclear cells (PBMCs) were separated by Ficoll-Paque density-gradient centrifugation (Eurobio, Courtaboeuf, France). Red cells and platelets were eliminated using a hypotonic solution and centrifugation.

### Cell Isolation

CD14^++^CD16^−^, CD14^++^CD16^+^, and CD14^dim^CD16^++^ subsets were FACS Aria sorted from PBMCs based on size morphology and CD14^++/dim^CD16^++/−^ expression for differentiation with IL-34 (Supplementary Figure 1E). Total CD14^+^ monocytes were isolated using a negative selection kit (Miltenyi Biotec., Bergisch Gladbach, Germany) for phosphorylation analysis, or by magnetic depletion (Dynabeads, Invitrogen) of CD3^+^, CD16^+^, and CD19^+^, then FACS Aria sorting of CD14^++^ cells for both RNA sequencing analysis and Treg expansion. CD8^+^ Tregs were obtained by enrichment of PBMCs in T cells (to 80% T cells) by magnetic depletion of CD19^+^, CD14^+^, and CD16^+^ and then sorting of CD3^+^CD4^−^CD45RC^low/−^ cells (Supplementary Figure 4A) using FACS ARIA II (BD Biosciences, Mountain View, CA, USA). Allogeneic APCs were isolated by magnetic depletion of CD3^+^ cells from PBMCs.

### Quantification of CSF-1R and PTPζ Signaling Pathway Activation

Freshly sorted CD14^+^CD16^−^ monocytes were plated at 1 × 10^6^ cells/ml in fetal bovine serum (FBS)-free RPMI 1640 medium (1% penicillin-streptomycin, 1 mM glutamine, 1% NEAA, 10 mM Hepes, 1 mM sodium pyruvate) in low attachment round-bottomed 96-well plates (Perkin-Elmer, Inc., Waltham, MA, USA), and left untouched for 2 h before adding IL-34 or CSF-1 at a final concentration of 100 ng/ml. Analysis of the phosphorylation of AKT and ERK1/2 after 1, 3, 5, 10, and 15 min was performed by flow cytometry following the BD Biosciences Phosflow protocol, using the BD Cytofix Fixation buffer and BD Phosflow Perm Buffer III (BD Biosciences), as well as phospho-AKT (Ser473) and phospho-p44/42 MAPK (Erk1/2) (Thr202/Tyr204) primary goat antibodies (Cell Signaling Technology, Leiden, The Netherlands), and goat anti-rabbit IgG(H+L)-AF647 (Life Technologies, ThermoFisher Scientific) secondary antibody.

### Differentiation of Monocytes and Expansion of Tregs

Monocytes were seeded at 1 × 10^6^ cells/mL in complete RPMI 1640 medium supplemented with 10% FBS and IL-34 (2 nM, eBiosciences, ThermoFisher Scientific, Waltham, MA, USA) or CSF-1 (2 nM, R&D Systems, Bio-techne, Minneapolis, MN, USA) and macrophages were harvested at day 6. M1 macrophages were obtained by supplementing the medium with granulocyte-macrophage colony-stimulating factor (GM-CSF, 10 ng/mL, Cellgenix, Freiburg, Germany) over 5 days and by addition of interferon-gamma (IFNγ, 1000 U/mL, Miltenyi Biotec) from day 5 until day 7 of culture. M2 macrophages were obtained by supplementing the medium with CSF-1 (25 ng/mL, R&D Systems Biotechne) for 5 days and by addition of IL-4 (20 ng/mL, Cellgenix) and IL-10 (20 ng/mL, R&D Systems Biotechne) from day 5 until day 7 of culture. Lipopolysaccharide (LPS, 100 ng/mL, Sigma Aldrich, Saint-Louis, MO, USA) was added in the culture for the last 24 h for cytokine dosage. Macrophages were harvested using Trypsin (TryPLE, Gibco, ThermoFisher Scientific) at day 7.

Allogeneic PBMCs were seeded at 1 × 10^6^ in 24-well plate in Iscove's modified Dulbecco's medium (IMDM), supplemented with 2 mM glutamine, 100 U/ml penicillin, 0.1 mg/ml streptomycin, and 5% human AB serum with IL-34- or CSF-1- differentiated macrophages at a ratio of PBMCs:macrophages 5:1 and cultured for 14 days.

CD8^+^CD45RC^low/−^ Tregs were seeded at 5 × 10^5^ cells/cm^2^/500 μl in flat-bottom plates coated with anti-CD3 mAb (1 μg/mL, OKT3, hybridoma from the European Collection of Cell Culture), in complete RPMI 1640 medium supplemented with 10% FBS, IL-2 (1,000 U/mL, Proleukin, Novartis), IL-15 (10 ng/mL, Miltenyi Biotec) and soluble anti-CD28 mAb (1 μg/mL, CD28.2, hybridoma from the European Collection of Cell Culture) in the presence of IL-34-differentiated macrophages or allogeneic APCs irradiated (35 Gy) at 1:4 Treg:IL-34-macrophage or APC ratio. CD8^+^ Tregs were stimulated again using anti-CD3 and anti-CD28 mAbs at day 7 of culture and IL-2 and IL-15 were freshly added at days 0, 2, 4, 7, 10 and 12.

### Monoclonal Antibodies and Flow Cytometry

Antibodies used are listed in Table 1 and Supplementary Table 1. For analysis of intracellular cytokines, Tregs were incubated with PMA, ionomycin, and brefeldine A (10 μg/ml) for 4 h before staining. Fc receptors were blocked (BD Biosciences) before staining and cells were permeabilized with a Fix/Perm kit (Ebiosciences).

**Table 1:** List of antibodies used.

**Marker**	**Clone**	**Provider**
CD14	M5E2	BD Biosciences
CD16	3G8	BD Biosciences
CD115	9-4D2-1E4	BD Biosciences
PTPζ	Polyclonal	Bioss
CD138	MI15	BD Biosciences
CD3	SK7	BD Biosciences
CD4	RPA-T4	BD Biosciences
CD8	RPA-T8	BD Biosciences
CD25	M-A251	BD Biosciences
CD45RC	MT2	IQProduct
CD19	HIB19	BD Biosciences
CD56	B159	BD Biosciences
CD335	9E2/Nkp46	Biolegend
CD86	2331	BD Biosciences
CD80	L307.4	BD Biosciences
CD40	5C3	BD Biosciences
CD206	19.2	BD Biosciences
CD169	7-239	BD Biosciences
CD163	GHI/61	BD Biosciences
CD209a	DCN46	BD Biosciences
CD36	HIT2	BD Biosciences
CD1a	HI149	BD Biosciences
IL-34	578416	R&D System
TGFβ1	TW4-9E7	BD Biosciences
FOXP3	259D/C7	BD Biosciences
IFNγ	B27	BD Biosciences
Tbet	O4-46	BD Biosciences
GITR	REA841	Miltenyi Biotec
PD-1	EH12.1	BD Biosciences
CD127	hIL-7R-M21	BD Biosciences
CD28	CD28.2	BD Biosciences
CD27	M-T271	BD Biosciences
CD45RA	HI100	BD Biosciences
HLA-DR	L243	BD Biosciences
CD154	TRAP1	BD Biosciences
TRAIL	RIK-2	BD Biosciences
CD103	Ber-ACT8	BD Biosciences
hCD45	HI30	BD Biosciences
mCD45	30-F11	BD Biosciences
Phospho-Akt (Ser473)	D9E	Cell Signaling Technology
Phospho-p44/42 MAPK (Erk1/2) (Thr202/Tyr204)	D13.14.4E	Cell Signaling Technology

Fluorescence was measured with LSR II or Canto II cytometers (BD Biosciences) and analyzed with FLOWJO software (Tree Star, Inc., Ashland, OR, USA).

### ELISA

IL-10 and IL-12p40 were quantified in the supernatant of monocytes cultured for 6 days as well as control M1 macrophages, and both were stimulated for the last 24 h with LPS at 100 ng/ml using Human IL-10 ELISA Set and Human IL-12p40 ELISA Set performed according to manufacturer's instructions (BD Biosciences).

### DGE-RNA Sequencing

CD14^++^CD16^−^ monocytes were sorted by FACS Aria and lysed in RLT Buffer (Qiagen). RNeasy-Mini Kits (Qiagen) were used to isolate total RNA that was then processed for RNA sequencing. A protocol of 3′ Digital Gene Expression (DGE) RNA-sequencing was performed as previously described (13). Library was run on an Illumina NextSeq 550 high-output (2 × 75 pb) (Genom'IC platform, Cochin Institute, Paris). Reads 1 encode for well-specific barcodes and unique molecular identifiers (UMIs) whereas Reads 2 encode for 3' mRNA sequences and were aligned to human genome reference (hg19). Count matrix was generated by counting sample-specific UMI associated with genes for each sample. Differentially expressed genes between conditions were calculated using R package Deseq2 (Bioconductor) by first applying a regularized log transformation (rlog). Genes with adjusted *p*-value inferior to 0.05 were considered as differentially expressed. Heatmaps were generated by scaling and center genes expression. Finally, a volcano plot was designed by plotting -Log10 of adjusted *p*-value in function of log2 Fold Change; highlighted genes correspond to differentially expressed genes. The accession number for DGE-RNA sequencing raw data and processed data is GEO: GSE151194.

### Single Cell RNAseq Analysis

An online public dataset of 10X genomics (https://support.10xgenomics.com/single-cell-gene-expression/datasets/3.0.2/5k_pbmc_v3_nextgem) was used to analyze gene expression of SDC1 (CD138), PTPRZ1 (PTPz) and CSF-1R in human PBMCs. Data were processed with “Seurat” package (version 3.1.3) in R software (RStudio, Inc., Boston). To eliminate unwanted cells (debris and doublets), cells with fewer than 200 genes or more than 4,000 genes were excluded. Then, cells with more than 10% of mitochondrial genes were excluded from the downstream analysis. Single cell transcriptomes were first normalized (log normalization) and then scaled. The most variable genes were found according to the variance stabilizing transformation (vst) method and were used to perform Principal Component Analysis (PCA). Clustering was performed on the first nine principal components, and hPBMC subsets were characterized according to expression of common membrane markers. Finally, a supervised analysis was performed to classify CD14^++^CD16^−^, CD14^++^CD16^+^, and CD14^dim^CD16^++^ monocytes.

### Immune Humanized Mouse aGVHD Model

This study was carried out according to permit numbers APAFIS 3168 from the Ministry of Research. Eight to twelve-week-old NOD/SCID/*Il2r*γ^−/−^ (NSG) mice were bred in our own animal facilities in SPF conditions (accreditation number C44-278). 1.5 × 10^7^ human PBMCs were intravenously injected in 1.5 Gy-irradiated NSG mice the day before, as previously described (14, 15). Human PBMCs were monitored in blood and GVHD development was evaluated by body weight loss (14, 15). Human recombinant IL-34 (0.4 or 0.8 mg/kg/2.5 d for 20 days; from eBiosciences) and/or anti-human CD45RC mAbs (0.8 mg/kg/2.5 d for 20 days, MT2 or ABIS-45RC clones) were injected intraperitoneally. PBMCs were i.v. injected alone or with Tregs in a range of PBMC:Treg ratio from 1:0.5 to 1:2.

### Statistical Analysis

Two-way repeated measure ANOVA was used to analyze mouse weight loss over time and Log Rank (Mantel Cox) test was used to analyze mouse survival. Friedman test with Dunn's multiple comparison test were used to compare monocyte frequency in PBMCs. Two-way ANOVA and Bonferroni post-test were used to analyze the survival of monocytes during the culture, phenotype of monocyte subsets and expanded Tregs. Mann Whitney *U*-test was used to compare the IL-10/IL-12p40 ratio in the supernatants of cultured macrophages.

## Results

### CSF-1R and PTPζ Are Both Expressed on CD14^++^ Monocytes and CSF-1R Is Also Expressed on FOXP3^+^ CD4^+^ and CD8^+^ Tregs

**Figure 1:**
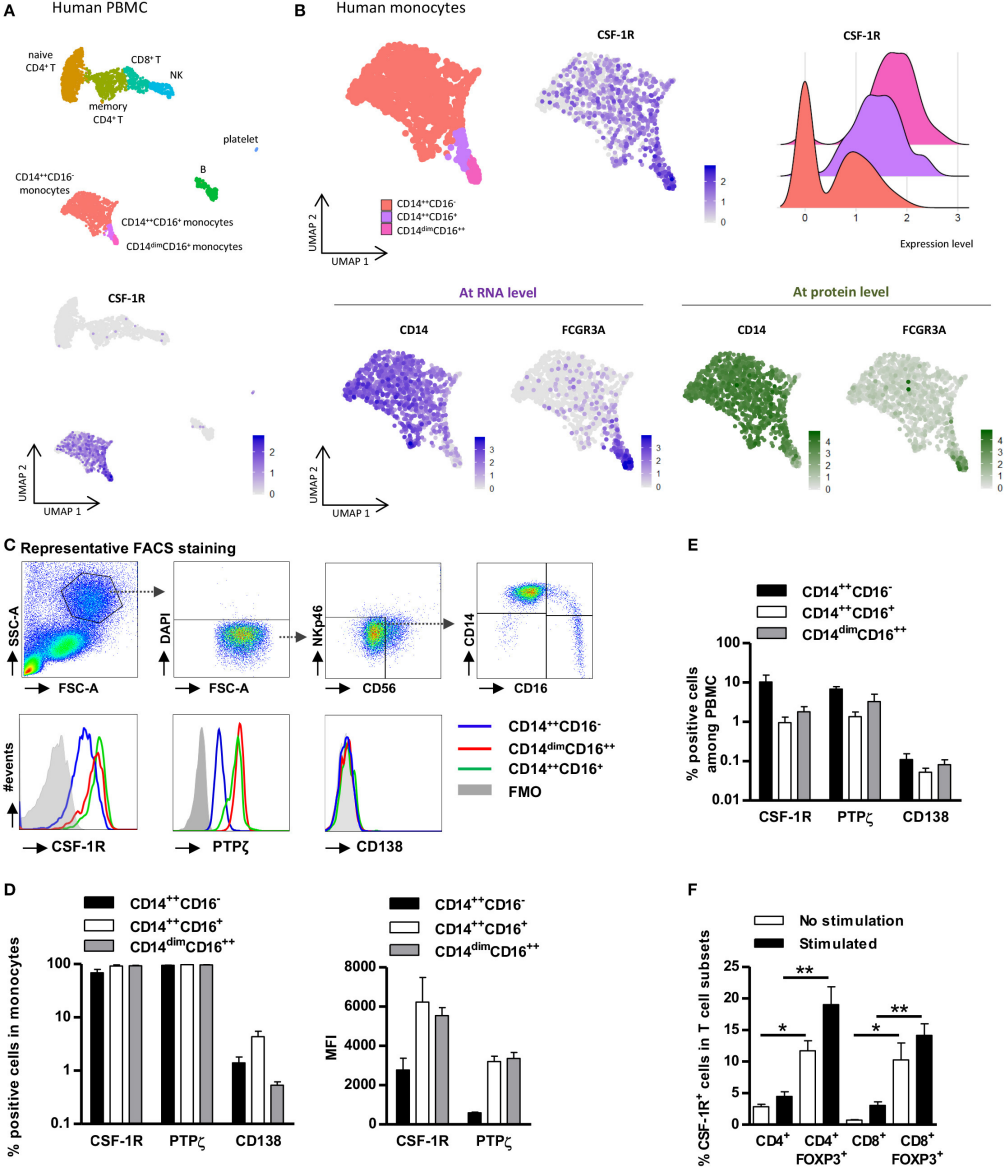
CSF-1R and PTPζ expression is restricted to monocytes and FOXP3^+^ Tregs. PBMCs were analyzed for CSF-1R expression at single cell transcriptional **(A,B)** and proteomic levels **(C–F)**. **(A)** Top: UMAP visualization of a public dataset of resting Human PBMC single cell RNA-seq from one healthy volunteer for which subsets of monocytes, T cells, B cells, and NK cells were identified by antibody staining. Bottom: CSF-1R expression in total PBMCs. One point represents one cell. Relative expression level is scaled from gray to dark blue. **(B)** Monocyte subsets were further subdivided based on RNA (RNAseq, bottom left) and protein expressions (CITEseq, bottom right) of CD14 (left) and FCGR3A (CD16) (right) summarized in the UMAP visualization (upper left), and subsets were analyzed for CSF-1R RNA expression (upper middle and right). One point represents one cell. Relative expression level is scaled from gray to dark blue (RNA expression) or from gray to dark green (protein expression). Upper Right: Violin plot representing the expression level of mRNA for CSF-1R in CD14^++^CD16^−^ monocytes (red), in CD16^++^CD14^dim^ monocytes (pink), and in CD14^++^CD16^+^ monocytes (purple). **(C)** Representative gating strategy for FACS analysis of CSF-1R, PTPζ, and CD138 expression in living (DAPI^−^) non-NK cells (CD56^−^NKp46^−^) CD14^++/dim^CD16^++/+/−^ cell subsets from PBMCs. Representative from three individuals. **(D)** Frequency (left) of CSF-1R, PTPζ, and CD138 expressing cells and expression level (MFI) of CSF-1R and PTPζ (right) in CD14^++/dim^CD16^++/+/−^ cell subsets. *n* = 3 individuals. **(E)** Frequency of CSF-1R^+^, PTPζ^+^, and CD138^+^ monocytes in total PBMCs. *n* = 3 individuals. **(F)** Frequency of CSF-1R expressing cells in stimulated (black) or not (white) FOXP3^+/−^ CD4^+^ or CD8^+^ T cells. *n* = 5 individuals. Mann Whitney tests, *p< 0.05, **p < 0.01.

We previously showed that IL-34 produced by FOXP3^+^ Tregs acted at least on human monocytes *in vitro* (12). To get a better overview of IL-34 action on the immune system, we analyzed the expression of its reported receptors CSF-1R (also called CD115), CD138 (also called SDC1), and PTPζ (also called PTPRZ1) on whole PBMCs using a public single cell RNAseq dataset (https://support.10xgenomics.com/single-cell-gene-expression/datasets/3.0.2/5k_pbmc_v3_nextgem). We observed that CSF-1R single cell mRNA expression was restricted to monocytes and not significantly expressed by resting T, B and NK cells (Figure 1A). Analysis of markers of non-classical (CD14^dim^CD16^++^), intermediate (CD14^++^CD16^+^), or classical (CD14^++^CD16^−^) monocytes/macrophages (16, 17) showed that CSF-1R was expressed in all three populations of monocytes (Figure 1B) with a higher expression in non-classical and intermediate monocytes. In contrast, CD138 and PTPzeta mRNA expression was not detectable in resting PBMCs (Supplementary Figures 1A,B). However, we were able to detect PTPζ protein expression in all monocyte subsets and we also confirmed that CSF-1R was expressed by all monocytes, and both with a higher expression level in non-classical monocytes (Figures 1C,D). Nevertheless, since CSF-1R^+^ and PTPζ^+^ classical monocyte frequency in PBMCs is much higher than CSF-1R^+^ and PTPζ^+^ intermediate and non-classical monocytes (Figure 1E), it suggests that IL-34 will mostly act on CD14^++^CD16^−^ monocytes.

To better comprehend whether IL-34 could act directly on Tregs, we further analyzed CSF-1R and PTPζ expression in total CD4^+^ or CD8^+^ T cells compared to FOXP3^+^ CD4^+^ or CD8^+^ Tregs (Figure 1F and Supplementary Figures 1C,D). We observed a significant expression of CSF-1R in non-stimulated FOXP3^+^ CD4^+^ and CD8^+^ Tregs compared to total CD4^+^ and CD8^+^ T cells, respectively (Figure 1F and Supplementary Figure 1C). The expression was even higher following stimulation, although it remains lower than on monocytes. We did not observe expression of PTPζ on Treg cells (Supplementary Figure 1D).

Altogether, these results suggest that IL-34 can act on CD14^++^ monocytes, likely through CSF-1R and PTPζ and on FOXP3^+^ Tregs through CSF-1R in PBMCs.

### IL-34 Preferentially Acts Through CD14^++^CSF-1R^+^PTPζ^+^ Monocytes to Induce Immunoregulation

**Figure 2:**
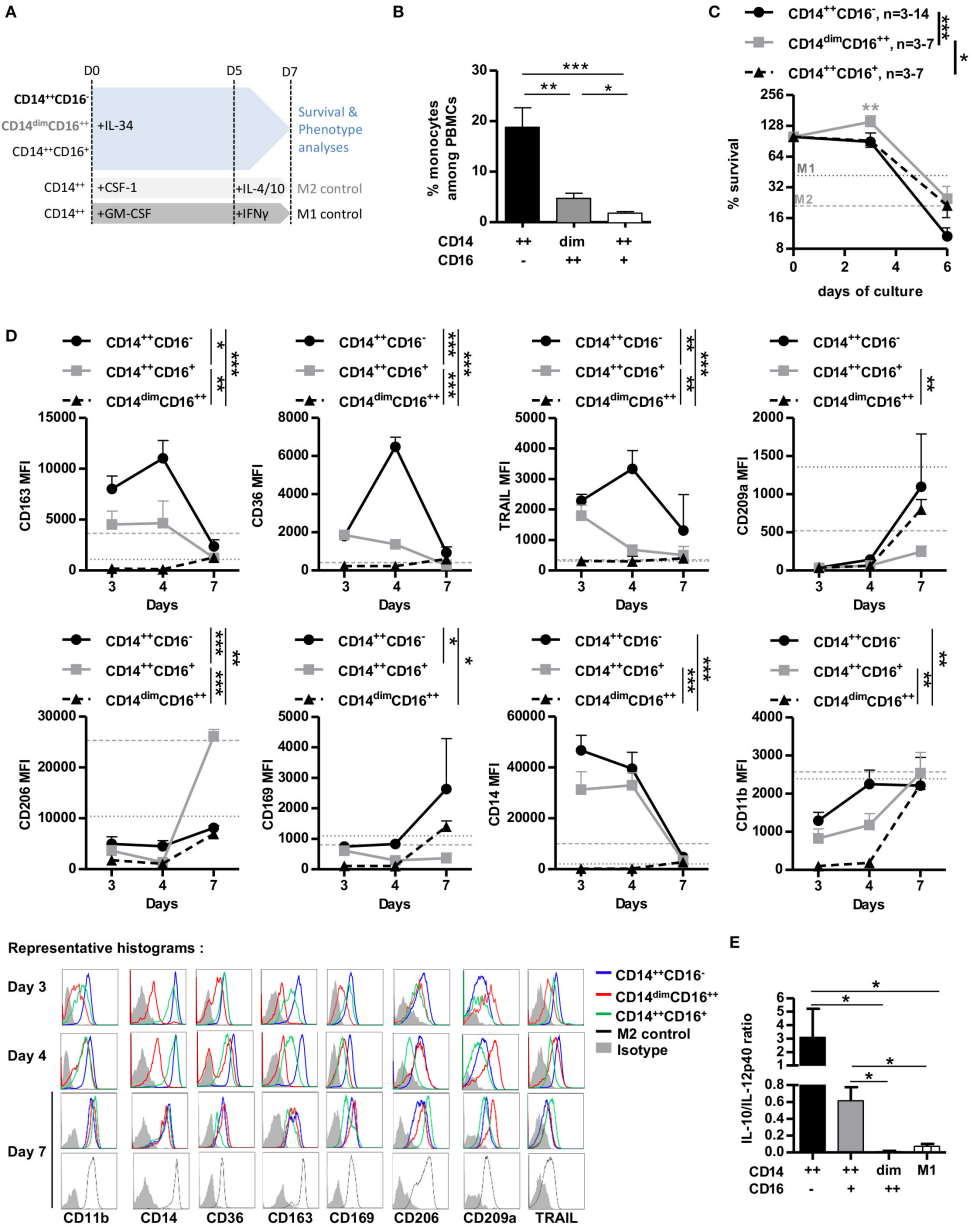
CD14^++^ monocytes are the main mediators of IL-34-induced immunoregulation. **(A)** Schematic depicting conditions and timing of supplementation in cytokines in monocyte cultures. LPS was added for the last 24 h for cytokine release analysis only. **(B)** Frequency of monocyte subsets in PBMCs of healthy individuals. *n* = 8 individuals. Mann Whitney tests, *p < 0.05, **p < 0.01, ***p < 0.001. **(C)** Living cell count over 6-days culture normalized to day 0 (=100%). *n* = 3–14 individuals. M1 (dark gray dotted line) and M2 (light gray dashed line) macrophages mean survival of three individuals after 7-days culture is shown. Two-way ANOVA and Bonferroni post-test. *p < 0.05, **p < 0.01, ***p < 0.001. **(D)** Monocyte subsets were cultured for 7 days in the presence of IL-34 and analyzed for surface marker expression. Top: Geometric mean of fluorescence +/– SEM out of three experiments is represented over time. M1 (dark gray dotted line) and M2 (light gray dashed line) macrophages mean of fluorescence of three individuals after 7-days culture is shown. Mann Whitney *U*-test, *p < 0.05, **p < 0.01, and ***p < 0.001. Bottom: Representative histograms of FACS staining. CD14^++^CD16^−^ (blue line), CD14^dim^CD16^++^ (red line), and CD14^++^CD16^+^ (green line). Isotypic control is shown in filled gray. **(E)** IL-10/IL-12p40 ratios secreted by LPS-activated macrophages were quantified in supernatants at day 6. *n* = 3–5 individuals. Mann Whitney *U*-test, *p < 0.05.

We and others have shown that IL-34 induces differentiation of human CD14^++^ monocytes into macrophages with regulatory properties (12, 18). However, we observed that CSF-1R and PTPζ expressions was higher on non-classical and intermediate than classical monocytes, thus we investigated in each of the three subpopulations the survival and maturation upon IL-34 treatment compared to M1- and M2-macrophages differentiated with GM-CSF+IFNγ or CSF-1+IL-4+IL-10, respectively, as controls (18, 19) (Figure 2A and cell sorting in Supplementary Figure 1E). Classical monocytes were largely predominant over intermediate and non-classical monocytes among PBMCs (about 18.8 vs. 4.7 vs. 1.8%, respectively, Figure 2B), and together with intermediate monocytes had a lower survival rate after 6-days culture than non-classical monocytes (10.6 vs. 24.7 vs. 21.2% for CD14^++^CD16^−^, CD14^dim^CD16^++^, and CD14^++^CD16^+^, respectively, Figure 2C). Comparing the phenotype, classical monocytes differentiated with IL-34 expressed higher levels than non-classical monocytes of M2-type markers CD163, CD36, CD169, CD206, CD14, and TRAIL (Figure 2D and Supplementary Figure 1F), displayed an anti-inflammatory cytokine secretion profile (Figure 2E), were isolated (vs. in clumps for non-classical differentiated monocytes) and displayed fewer dendrites under macroscopic observation (vs. intermediate and non-classical monocytes) (Supplementary Figure 1G). Intermediate monocytes had an intermediate phenotype, closer to classical than non-classical monocytes (Figures 2D–E). Interestingly, non-classical monocytes expressed high levels of the M2-associated marker CD209a after culture in the presence of IL-34 (Figure 2D). Finally, CD11b was more expressed in classical and intermediate monocytes, in accordance with previous observations (12, 20).

These results show that IL-34 is more efficient at inducing M2-like macrophages from classical and intermediate monocytes than non-classical monocytes and suggest that CD14^++^CSF-1R^+^PTPζ^+^ monocytes are the cells through which IL-34 induces immunoregulation.

### IL-34 Efficiently Induces Regulatory Macrophages From Classical Monocytes Expressing Different Genes Than CSF-1-Treated Macrophages

**Figure 3:**
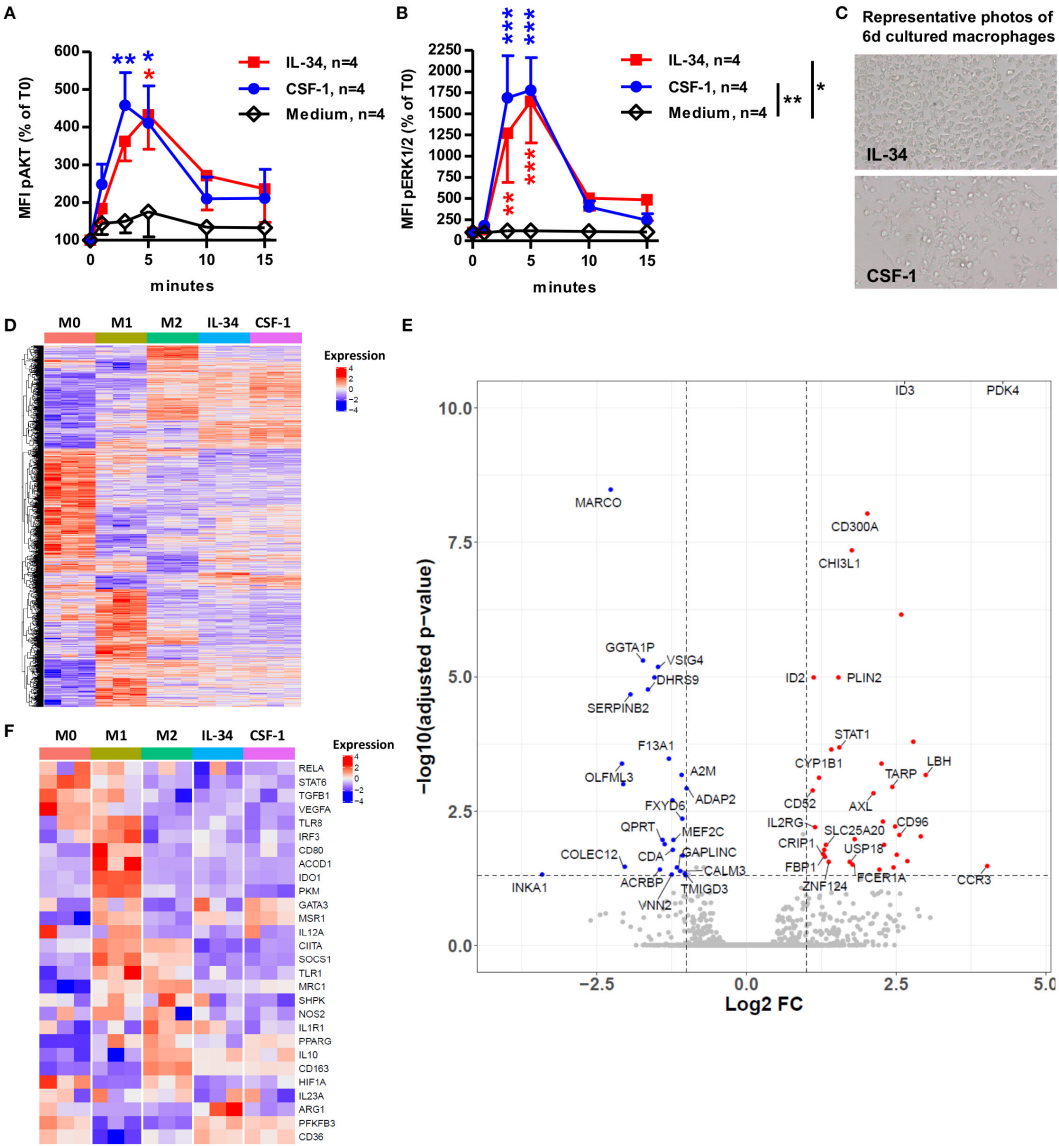
IL-34-induced macrophages display a transcriptome close, but not identical, to M2-type and CSF-1-induced macrophages. **(A,B)** CD14^++^ monocytes were cultured with IL-34 or CSF-1 for 1, 3, 5, 10, and 15 min and analyzed for phosphorylation of AKT **(A)** and ERK1/2 **(B)** by flow cytometry. Results are represented as a percentage of baseline levels (T0). *n* = 4 individuals. Two-way ANOVA and Bonferroni post-test compared to medium alone. *p < 0.05, **p < 0.01, ***p < 0.001. **(C)** Photos of CD14^++^ monocytes after 6 days of culture in the presence of IL-34 or CSF-1. X20 magnification. **(D–F)** CD14^++^ monocytes were cultured for 6 days with IL-34 or CSF-1 and analyzed by DGE-RNAseq for gene expression. **(D)** Expression levels of differentially expressed genes between each condition are presented as a heatmap. Each column represents one sample. Blue color represents low expressed genes and red color represents highly expressed genes. The color bar shows experimental conditions. M0 are freshly sorted monocytes. **(E)** Volcano plot highlighting overexpressed genes (on the right, red dots) and under-expressed genes (on the left, blue dots) in IL-34-differentiated macrophages as compared with CSF-1-differentiated macrophages. The *p*-value adjusted cut-off is 0.05. **(F)** Heatmap representing expression of M1 and M2 macrophage genes in samples. Gene expression was normalized with regularized log transformations (rlog) algorithm (Deseq2), center and scaled. Blue color represents low expressed genes and red color represents highly expressed genes. Supervised clustering was performed to order samples. The color bar corresponds to experimental conditions.

We further investigated the signal induced in CD14^++^CD16^−^ classical monocytes by IL-34 after binding the CSF-1R and PTPζ receptors in comparison to the signal induced by CSF-1 binding CSF-1R only. We observed a significant increase in the levels of phosphorylated AKT (Figure 3A) and ERK1/2 (Figure 3B) at 3 and 5 min following the addition of both IL-34 and CSF-1, compared to medium alone. CSF-1 induced non-significant slighter and higher levels of AKT and ERK1/2 phosphorylation compared to IL-34. After 6 days of culture, we observed morphological differences in the presence of IL-34 compared to CSF-1, with fewer dendrites and a more rounded morphology for IL-34-differentiated macrophages (Figure 3C), suggesting a difference in the phenotype of the differentiated macrophages. To further understand the similarities and differences of the IL-34 vs. CSF-1 induced macrophages, we performed a 3' digital gene expression RNA-sequencing (DGEseq) and compared freshly isolated CD14^++^ monocytes (M0), 6-days differentiated macrophages in the presence of GM-CSF+IFNγ (M1), CSF-1+IL-4+IL-10 (M2), IL-34 alone, or CSF-1 alone (Figures 3D–F). Transcriptomic clustering (Figure 3D), principal component (Supplementary Figure 2A), and Pearson correlation (Supplementary Figure 2B) analyses highlighted the transcriptional changes following differentiation and indicated clear divergence between CD14^++^ monocytes (M0) and M1-macrophages vs. all other groups and a clear convergence between M2-macrophages, IL-34-macrophages, and CSF-1-macrophages (Figure 3D and Supplementary Figures 2A,B). Further analysis of significant genes differentially expressed between IL-34 and CSF-1-macrophages revealed differential expression of 61 genes, with an upregulation of the expression of some interesting genes. Among those genes, we identified *PDK4*, a metabolic checkpoint for macrophage differentiation, *CHI3L1*, a carbohydrate-binding lectin that may play a role in tissue remodeling and cell capacity to respond to the environment involved in regulating Th2 cell responses and M2 macrophages differentiation, *FCER1A*, a receptor expressed by DCs that can play pro- or anti-inflammatory roles, and *CD300A*, a cell membrane receptor that contains classical ITIM motifs and negatively regulates Toll-like receptor (TLR) signaling mediated by MYD88 through the activation of PTPN6 and of macrophages in animal models (21). In contrast, we observed a down-regulation of *MARCO*, a marker of pro-inflammatory macrophages in IL-34-differentiated macrophages compared to CSF-1-differentiated macrophages (Figure 3E and Supplementary Figure 2C). Interestingly, further analysis of typical markers of macrophages (22) showed a preferential expression of some genes, such as *arginase-1* (*ARG1*) in IL-34 macrophages, compared to CSF-1, M1, and M2-differenciated macrophages, or *IDO1* that was found expressed only in M1 macrophages (Figure 3F). Other genes, like *IL-10*, in contrast were expressed by M2, IL-34, and CSF-1 macrophages.

Thus, IL-34 induced a high activation of monocytes through CSF-1R, subsequently inducing macrophages with a specific signature conferring regulatory/anti-inflammatory functions.

### IL-34 Prolongs Survival in a Model of Humanized Acute GVHD Through Treg Expansion Rather Than Generation of Induced Treg From Naive T Cells

**Figure 4:**
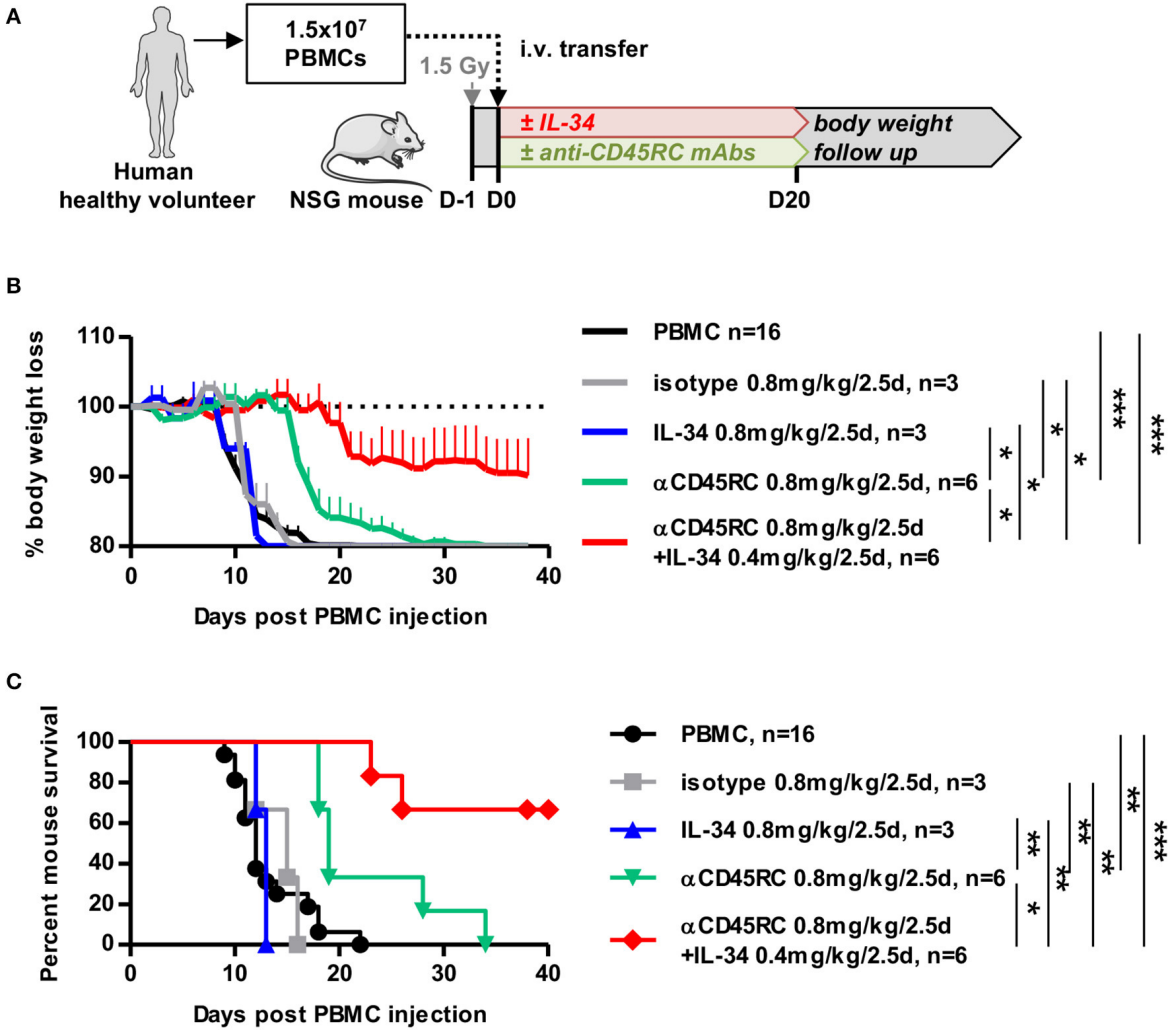
IL-34 in combination with depletion of naive cells prolongs survival in an acute GVHD humanized model. **(A)** Schematic depicting the GVHD model in humanized mice. NSG mice were injected with human PBMCs, treated or not with IL-34 protein and/or anti-CD45RC mAbs for 20 days, and followed for body weight loss. **(B)** Evolution of mouse body weight over time, normalized to the weight before the injection of PBMCs (D0), after no treatment (black line), IL-34 treatment (blue line), anti-CD45RC mAb treatment (green line), isotype Ig control treatment (gray line), and dual IL-34 + anti-CD45RC mAb treatment (red line). *n* = 3–16. Mean ± SEM is represented. Two Way repeated measure ANOVA, *p < 0.05, ***p < 0.001. **(C)** Percentage of mouse survival over time. *n* = 3–16. Log Rank (Mantel-Cox) test, *p < 0.05, **p < 0.01, ***p < 0.001.

We highlighted previously that IL-34 treatment in a model of cardiac allo-transplantation resulted in the induction of highly suppressive Tregs through M2-like macrophages *in vivo* in rat and *ex vivo* in human (12). However, whether IL-34-induced Tregs resulted from the expansion of natural pre-existing Tregs or from newly converted Tregs from naive/effector T cells was not clear. Thus, we used an anti-CD45RC antibody (mAb) that specifically eliminates naive and precursor effector T cells (Teff) (13) and depleted *in vivo* CD45RC^high^ Teff cells using a short-term course of anti-CD45RC mAb (as we previously described) in immunodeficient NOD/SCID/IL2rγ^null^ (NSG) mice injected with human PBMCs with or without IL-34 administration (Figure 4A and Supplementary Figures 3A,B). We observed that low-dose anti-CD45RC mAb treatment significantly delayed GVHD occurrence from 13.25 ± 0.9 days (mean survival) to 22.67 ± 2.7 days (Figures 4B,C). Although, low dose IL-34 treatment every 2.5 days at 0.8 mg/kg over 20 days was not sufficient to delay GVHD; IL-34 recombinant protein in combination with anti-CD45RC mAb therapy synergized and inhibited GVHD mortality in 66% of mice (Figures 4B,C). Analysis of mouse blood showed an efficient depletion of CD45RC^high^ cells during the anti-CD45RC mAb treatment with no impact on the engraftment of other human PBMC subsets (Supplementary Figure 3).

These results suggest that Teff cell depletion in combination with IL-34 administration can more efficiently control immune responses.

### IL-34 Induces, More Efficiently Than CSF-1, FOXP3^+^ Tregs Which Delay Xenogeneic GVHD

**Figure 5:**
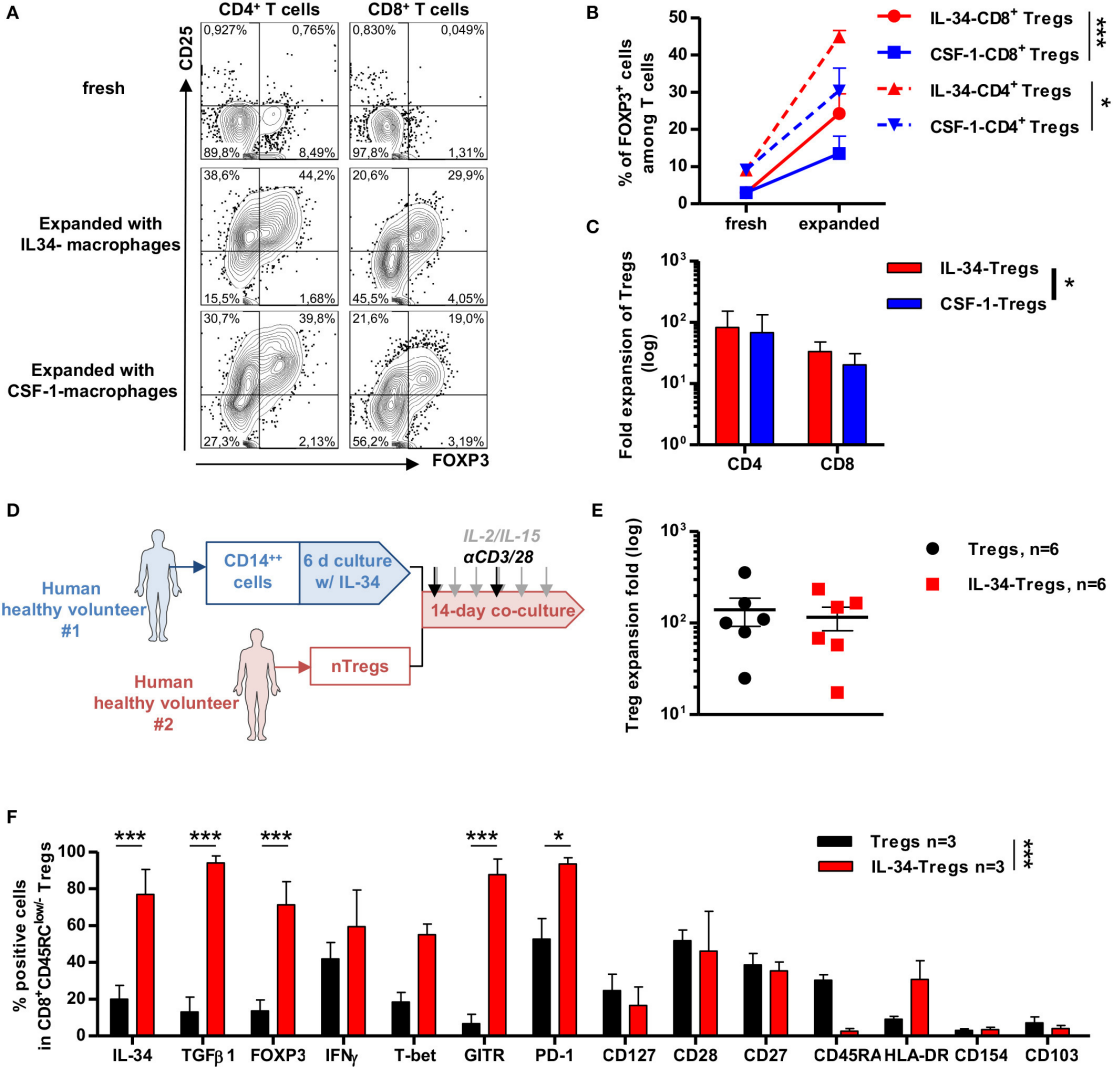
IL-34 potentiates differentiation of CD4^+^ and CD8^+^ FOXP3^+^ *in vitro* more effectively than CSF-1. **(A)** Representative FACS staining of CD25 and FOXP3 expression in CD4^+^ (left) and CD8^+^ (right) T cells after 14-day culture of PBMCs with either IL-34-differentiated macrophages (middle) or CSF-1-differentiated (bottom) macrophages compared to fresh cells (upper). **(B)** Frequency of FOXP3 positive cells in CD4^+^ (dotted lines) and CD8^+^ (solid line) T cells before and after expansion with IL-34- (red lines) or CSF-1- (blue lines) differentiated macrophages. Two-way ANOVA, *p < 0.05, ***p < 0.001. **(C)** CD4^+^ and CD8^+^ Tregs count harvested after 14 days of culture with IL-34- (red bars) or CSF-1- (blue bars) differentiated macrophages in fold expansion. Two-way ANOVA, *p < 0.05. **(D)** Schematic depicting cell culture. CD14^++^ monocytes were sorted from a healthy volunteer (HV#1), cultured for 6 days in the presence of IL-34, then added to CD8^+^CD45RC^low/−^ Tregs harvested from another healthy volunteer (HV#2) and 14-day cultured in the presence of a polyclonal stimulation once per week and IL-2 + IL-15 supplementation three times per week. **(E)** Treg cell count harvested after 14 days of culture with IL-34-differentiated macrophages or freshly isolated APCs normalized to Treg cell count seeded at day 0. **(F)** IL-34-Tregs (red bars) were analyzed by flow cytometry for Treg-associated marker expression as compared to before expansion (fresh cells, black bars). *n* = 3 individuals. Two-way ANOVA and Bonferroni post-test, *p < 0.05, ***p < 0.001.

We have previously shown that long-term tolerance in an allogeneic transplant model in rats treated with IL-34 was due to CD4^+^ and CD8^+^ Tregs that can control transplant rejection upon adoptive cell transfer (12). We also showed that human Tregs expanded from total PBMCs in the presence of IL-34-differentiated allogeneic macrophages suppressed immune response *in vitro* more potently than Tregs generated with monocytes in the absence of IL-34 (12). However, we did not assess whether this effect was comparable between IL-34 and CSF-1 or how these Tregs generated with IL-34 *in vitro* behaved *in vivo*. To do so, CD14^++^ monocytes from healthy volunteers were cell-sorted and differentiated in the presence of IL-34 or CSF-1 for 6 days and then added to allogeneic PBMCs for 14 days in the presence of IL-2 and IL-15 and a polyclonal stimulation. We thus observed that in both CD4^+^ and CD8^+^ T cells, IL-34 increased more efficiently the frequency of CD25^+^FOXP3^+^ Tregs than CSF-1 (Figures 5A,B), and this increase was even more significant for FOXP3^+^CD8^+^ Tregs for which CSF-1 had little effect (Figure 5B). In addition, analysis of the number of CD4^+^ and CD8^+^ Tregs following a 14-day expansion in the presence of IL-34-differentiated macrophages demonstrated a higher number of total Tregs (both CD4^+^ and CD8^+^) compared to expansion in the presence of CSF-1-differentiated macrophages (Figure 5C).

We previously reported that polyclonal or chimeric antigen receptor (CAR)-modified CD8^+^ Tregs can be efficiently expanded *in vitro* and control xenogeneic GVHD *in vivo* (14, 15). Given the efficacy of IL-34 to preferentially expand FOXP3^+^ Tregs, we then assessed the therapeutic benefit of using IL-34 in the CD8^+^ Treg expansion process for cell therapy. For this, we cultured naive CD8^+^CD45RC^low/−^ Tregs from PBMCs for 14 days in the presence of macrophages differentiated from CD14^++^ monocytes by IL-34 compared to freshly isolated APCs, IL-2, and IL-15 cytokines, and a low polyclonal anti-CD3/anti-CD28 mAbs stimulation (Figure 5D and Supplementary Figure 4A). We obtained more than an 100-fold expansion of CD8^+^ Tregs with either IL-34-differentiated macrophages (named IL-34-Tregs) or untreated macrophages (named Tregs) (Figure 5E). After expansion, IL-34-Tregs were highly enriched in FOXP3^+^ cells, expressed higher levels of surface markers commonly related to CD4^+^ and CD8^+^ Tregs, such as GITR and PD-1, and cytokines such as TGFβ, IFNγ, and IL-34 that we have demonstrated as being mediators of CD8^+^ Treg-suppressive activity (23, 24) (Figure 5F and Supplementary Figure 4B).

**Figure 6:**
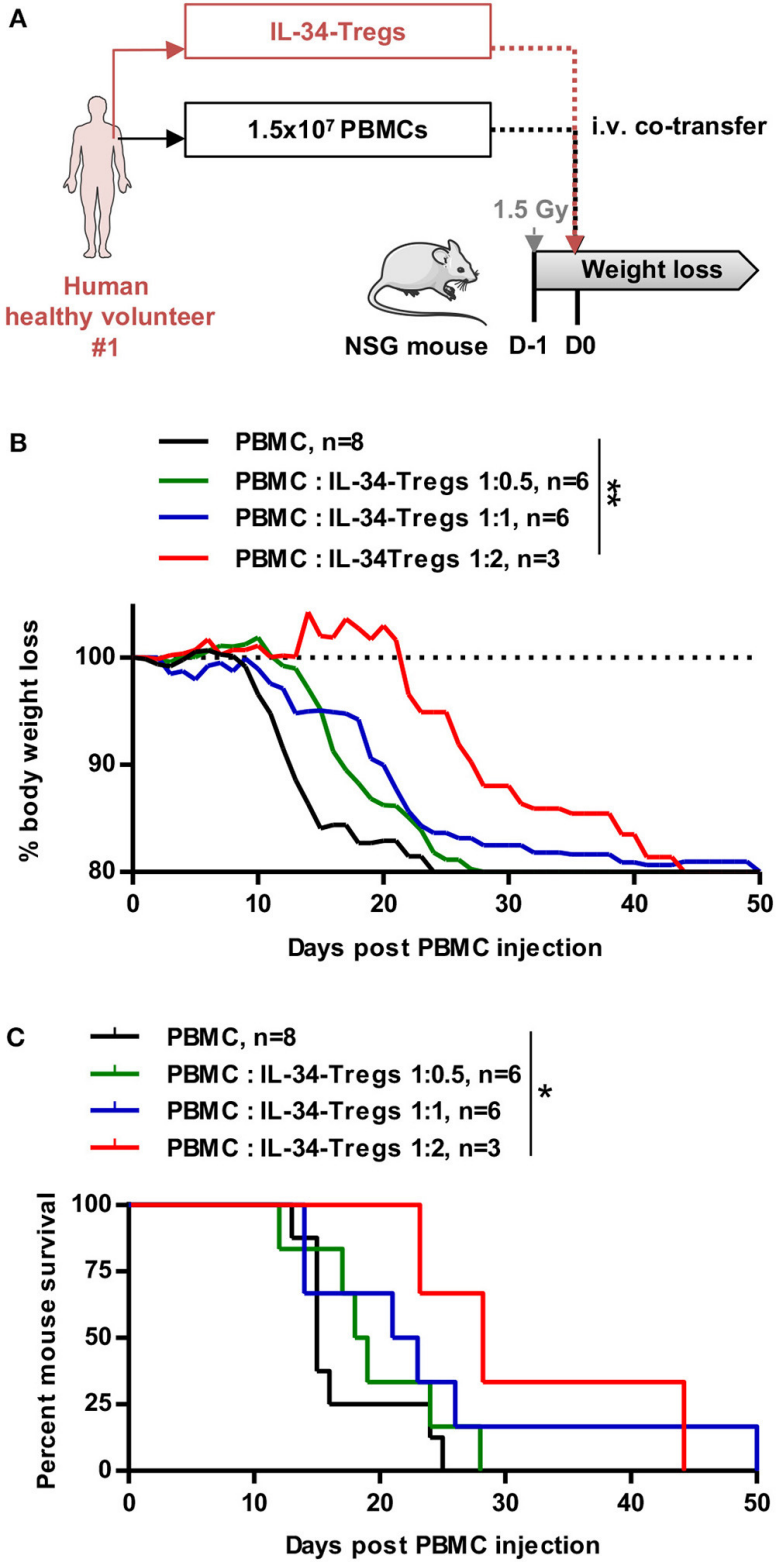
Cell therapy with IL-34-expanded CD8^+^ Tregs delays aGVHD. **(A)** Schematic depicting treatment of mice in the model of xenogeneic GVHD. PBMCs injected are syngeneic to the expanded Tregs co-injected. **(B)** Mouse body weight follow-up and **(C)** mouse survival after PBMC injection (D0) with or without Tregs expanded in the presence of IL-34-differentiated macrophages in a range of PBMC:Tregs ratio. *n* = 3–8. **(B)** Two-way RM ANOVA, **p < 0.01. **(C)** Log Rank (Mantel Cox) test. *p < 0.05.

Finally, we assessed the suppressive function of IL-34-Tregs *in vivo* in a xenogeneic model of acute GVHD (Figures 6A–C). NSG mice were first injected with human PBMCs to induce a xenogeneic acute GVHD and were either treated or not with IL-34-Tregs in a range of PBMC:Treg ratios (Figures 6A–C and Supplementary Figures 4C,D). We observed that IL-34-Tregs significantly delayed body weight loss (Figure 6B) and mouse survival (Figure 6C) in a dose-dependent manner compared to the control group.

Altogether, these results demonstrate that IL-34 is beneficial for FOXP3^+^ Treg expansion *ex vivo* and that CD8^+^ Tregs expanded with IL-34 can control graft rejection in a dose-dependent manner.

## Discussion

**Figure 7:**
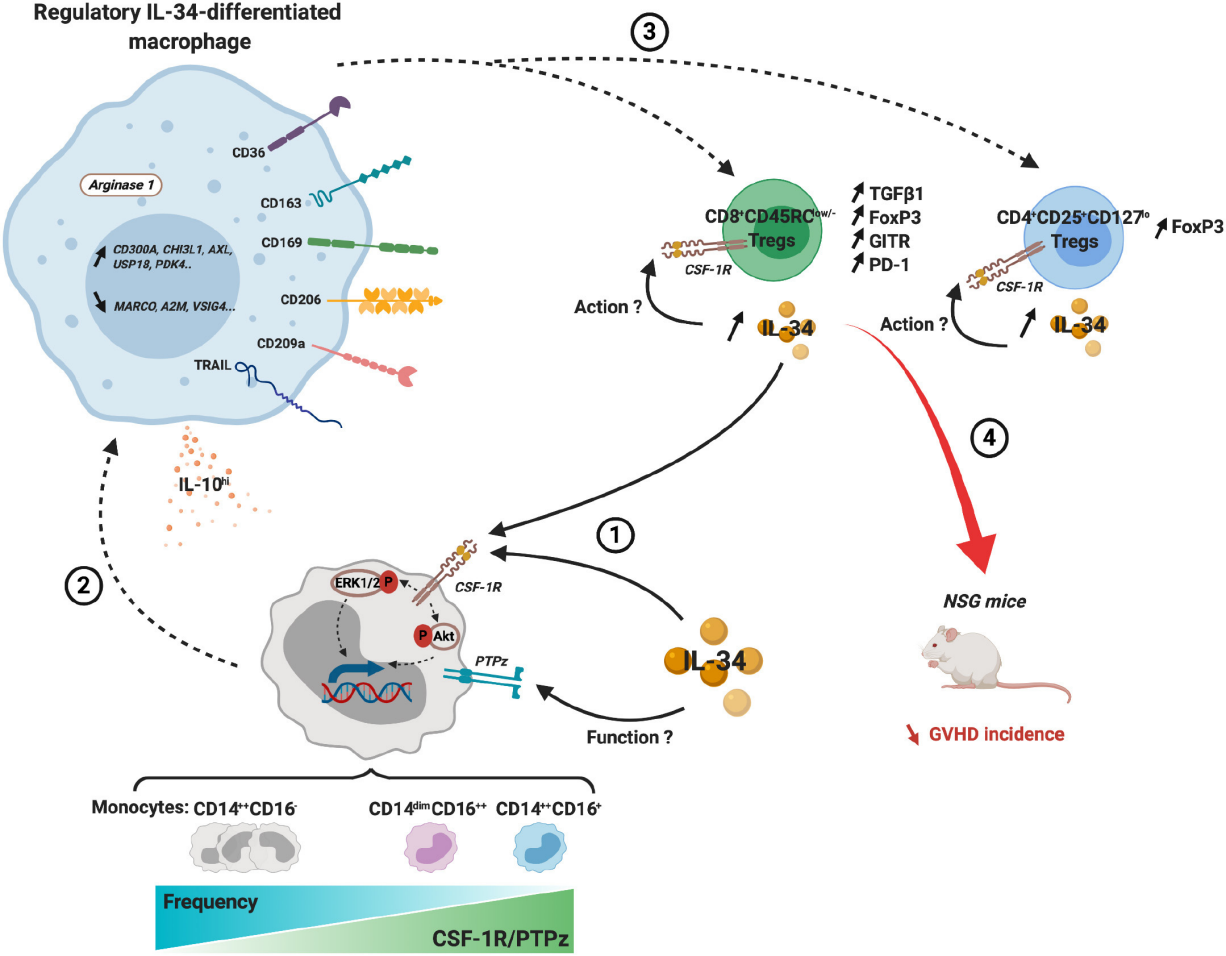
Integrated scheme of the regulatory actions of IL-34-differentiated macrophages and their ability to potentiate FOXP3^+^ Tregs. (1) IL-34 exogenously administered or from endogenous sources, such as from Treg, acts through CSF-1R to preferentially differentiate classical and intermediate monocytes into regulatory macrophages (2). (3) IL-34-differentiated macrophages expand and enhance the suppressive phenotype of both CD4^+^CD25^+^CD127^low^ and CD8^+^CD45RC^low/−^ Tregs. IL-34 secretion by Tregs maintains and increases the regulatory loop and can act in an autocrine fashion on Tregs. (4) In GVHD in NSG mouse, expanded CD8^+^ Tregs efficiently delay GVHD incidence. Dashed arrow, induction; solid arrow, binding.

Altogether, we have demonstrated that IL-34-treated CD14^++^CSF-1R^+^PTPζ^+^ monocytes were differentiated into pro-tolerogenic macrophages with a specific signature able to efficiently expand and potentiate FOXP3^+^ Tregs *in vitro* and *in vivo* to control anti-donor immune responses (Figure 7).

We found the expression of CSF-1R and PTPζ mostly on CD14^++^ classical and intermediate monocytes, although we found a more significant expression of both receptors on non-classical CD16^++^ monocytes (16, 25, 26). As for CSF-1, IL-34 could polarize all three subtypes of monocytes into type 2 (M2) macrophages depending on the environment (27). Non-classical macrophages in particular play an important role in the control of immune responses and have also been associated with wound-healing and resolution of inflammation in damaged tissues (28). PTPζ expression was mostly reported in the brain and, more recently, in the kidney (11, 29), while its expression on monocytes has only been suggested by western blotting (30); thus, our study confirms that both CSF-1R and PTPζ are expressed at the protein level by monocytes, suggesting that IL-34 action on monocytes through both PTPζ and CSF-1R could explain the differential effect compared to CSF-1. The intracellular signaling through PTPζ in monocytes still needs to be analyzed. The differential effects of IL-34 and CSF-1 can also be explained by the different binding characteristics and signaling through the CSF-1R that are discussed below.

We did not observe CSF-1R and PTPζ expression on resting total T cells, including Tregs, in single cell RNAseq data analysis of total PBMCs, probably because of the low frequency of Tregs and the low frequency of CSF-1R in Tregs compared to monocytes. However, using antibody staining, we were able to find a low expression of the protein CSF-1R on resting CD4^+^ and CD8^+^ FOXP3^+^ Tregs and upon stimulation this expression was significantly increased on activated CD4^+^ and CD8^+^ FOXP3^+^ Tregs. Thus it is possible that IL-34 acts directly on Treg polarization as TGFβ and IL-2, or on Treg function, in addition to acting through monocytes (31), and this will need to be further investigated.

Surprisingly, we did not observe any expression of CSF-1R in expanded FOXP3^+^ Tregs (data not shown), suggesting a transient expression of CSF-1R in Tregs upon activation and a narrow window for IL-34 to act directly on those cells. This further suggests a synergistic effect of IL-34 on monocytes and recently activated Tregs that supports the therapeutic strategy based on a short course treatment with IL-34 to induce tolerogenic monocytes and Tregs right after an immune challenge.

Although IL-34 and CSF-1 bind to the same receptor, CSF-1R, on the same cells, IL-34 can also act through PTPζ binding on monocytes, resulting in a different potential to induce FOXP3^+^ Tregs *in vitro*. They are several hypotheses to explain this important difference in their respective capacity to induce FOXP3^+^ Tregs (both CD4^+^ and CD8^+^). IL-34 and CSF-1 have very different sequences and structures, as well as a different affinity for CSF-1R (IL-34 has an affinity 34-fold superior to the one of CSF-1 for CSF-1R) (11, 32), and although they establish structurally similar binding to CSF-1R, it is possible that the subsequent signaling and the signaling and transcriptional pathways involved in the differentiation of the monocytes to macrophages and the phenotype of the differentiated macrophages are different (33, 34). The higher affinity of IL-34 to CSF-1R would suggest a more important signal transduction for IL-34 compared to CSF-1. In addition, the expression of PTPζ probably impacts on CSF-1R-signaling in monocytes. Whether PTPζ reinforces, weakens, fastens, or slows down the signal induced through CSF-1R needs further investigation. We observed that IL-34 and CSF-1 induced in a similar manner the phosphorylation of AKT and ERK1/2, two molecules involved in the signaling of both CSF-1R and PTPζ molecules. In addition, although we did not observe striking differences in the global transcriptomic profile of 6-days differentiated macrophages with either IL-34 or CSF-1, we did observe several functionally important genes differentially regulated. *Arginase-1* mRNA was highly and specifically increased in IL-34-differentiated macrophages. Arginase-1 degrades arginine, deprives NO synthase of its substrate, down-regulates nitric oxide production, and is one of the key factors by which regulatory macrophages or myeloid-derived suppressor cells suppress T cell responses (35, 36). Arginase-1^+^ macrophages also promote wound-healing and decrease T cell activation and induce it when tolerance is sought or when targeting Arginase-1 in cancer is the focus of current efforts (37, 38). We also observed significant upregulation of other genes, such as *PDK4*, a metabolic checkpoint for macrophage differentiation (39), *CHI3L1*, a marker of M2 macrophages (40), *FCER1A*, a receptor expressed by DCs and a few monocytes that can play pro- or anti-inflammatory roles (41, 42), or *CD300A*, a negative regulator of TLR signaling in IL-34-differentiated macrophages compared to CSF-1-differentiated macrophages, emphasizing the differences between IL-34 vs. CSF-1. Interestingly, we found several genes involved in macrophage phagocytosis downregulated [i.e., *MARCO* (43–45), *A2M* (46, 47), *VSIG4* (48), or *COLEC12* (49, 50)] or inhibitors of phagocytosis upregulated such as *CD300A* (51) in IL-34-differentiated macrophages compared to CSF-1-differentiated macrophages, suggesting a decreased capacity to phagocytes compared to CSF-1 (34), but this will need further investigation. Although we found a low number of genes differentially regulated between CSF-1- and IL-34-differentiated macrophages, these markers emphasized the difference of activity on CSF-1R and/or the impact of the exclusive binding of IL-34 on PTPζ. The role of these different genes on the observed promoting effect of IL-34 on Treg induction will also need further investigation.

The capacity of IL-34 to induce both CD4^+^ and CD8^+^ Tregs is interesting as it would suggest that both CD4^+^ and CD8^+^ FOXP3^+^ cells could be expanded together without cell sorting from total PBMCs and then the final product, enriched in both Treg subsets, could be administered subsequently *in vivo*. Maybe elimination of Teff and naive cells using anti-CD45RC mAbs, for example, as we showed *in vivo* that it was beneficial for IL-34-therapeutic potential, would also be beneficial *in vitro* in the expansion protocol (i.e., depletion of CD45RC^+^ cells by cell sorting). These results obtained with the anti-CD45RC mAb suggest that naive/effector T cells were not involved in IL-34 establishment of a control of immune responses and that Tregs were rather expanded cells than newly-generated cells. Although we cannot conclude on a direct effect of IL-34 on Tregs in this experiment, since human IL-34 does not cross-react on murine cells and can only act on human cells and since in this model of humanized mice, GVHD is mediated mostly by T cells, this suggests a direct effect of IL-34 on Tregs and will need to be the subject of further investigations. The synergy between IL-34 and anti-CD45RC mAb also suggests that *in vivo* IL-34 efficacy may be limited by Teff cells. Although the synergistic capacity of CD4^+^ and CD8^+^ Tregs is not yet clear, both subsets could show complementary effects and it could be beneficial to administer them together to patients (24). IL-34 could also be used *in vivo* together with Treg cell therapy to promote the persistence and the function of the induced Tregs, as is done with low-dose IL-2 or rapamycin (52, 53), by enrichment of the environment with tolerogenic macrophages and by direct action on Tregs. We have tested *in vivo* the FOXP3^+^CD8^+^ Tregs induced in the 14-day *ex vivo* expansion in a model of xenogeneic GVHD in immune-humanized mice, and we have observed a similar protective potential of the Tregs compared to what we have previously demonstrated using polyclonally expanded CD8^+^ Tregs (14). Thus, it suggests that efficient Tregs were expanded, even from total PBMCs as a starting material, which shows similar protection compared to Tregs expanded without IL-34. Thus, an important advantage of using IL-34 would be the co-expansion of CD4^+^ and CD8^+^ FOXP3^+^ Tregs from total PBMCs. Also, this suggests that upon improvement of this protocol, with for example selective effector T cell depletion before expansion, it could result in improved protection.

Altogether, our results highlight the potential of IL-34 to favor the development of FOXP3^+^ Tregs and suggest that this cytokine should be further considered for *in vitro* use or *in vivo* therapy.

## Data Availability Statement

The datasets presented in this study can be found in online repositories. The names of the repository/repositories and accession number(s) can be found in the article/Supplementary Material.

## Ethics Statement

The animal study was reviewed and approved by Ministry of Research. Blood from healthy individuals was obtained at the Etablissement Français du Sang (Nantes, France). Written informed consent was provided according to institutional guidelines.

## Author Contributions

CG and IA contributed conception and design of the study. CG, SB, and IA wrote sections of the manuscript. SB, AF, CS, AS, and NV performed experiments and analyzed data. All authors contributed to manuscript revision, read, and approved the submitted version.

## Conflict of Interest

CG, IA, and SB have patents on IL-34 that are pending and are entitled to a share in net income generated from the licensing of these patent rights for commercial development. The remaining authors declare that the research was conducted in the absence of any commercial or financial relationships that could be construed as a potential conflict of interest.
