# Supplementary material for: IL-34 Actions on FOXP3+ Tregs and CD14+ Monocytes Control Human Graft Rejection
Source: Front Immunol. 2020 Aug 11;11:1496. doi: 10.3389/fimmu.2020.01496 (PMC7431608; doi:10.3389/fimmu.2020.01496)
Supplement: Supplementary file 2 [file Data_Sheet_1.docx]

**
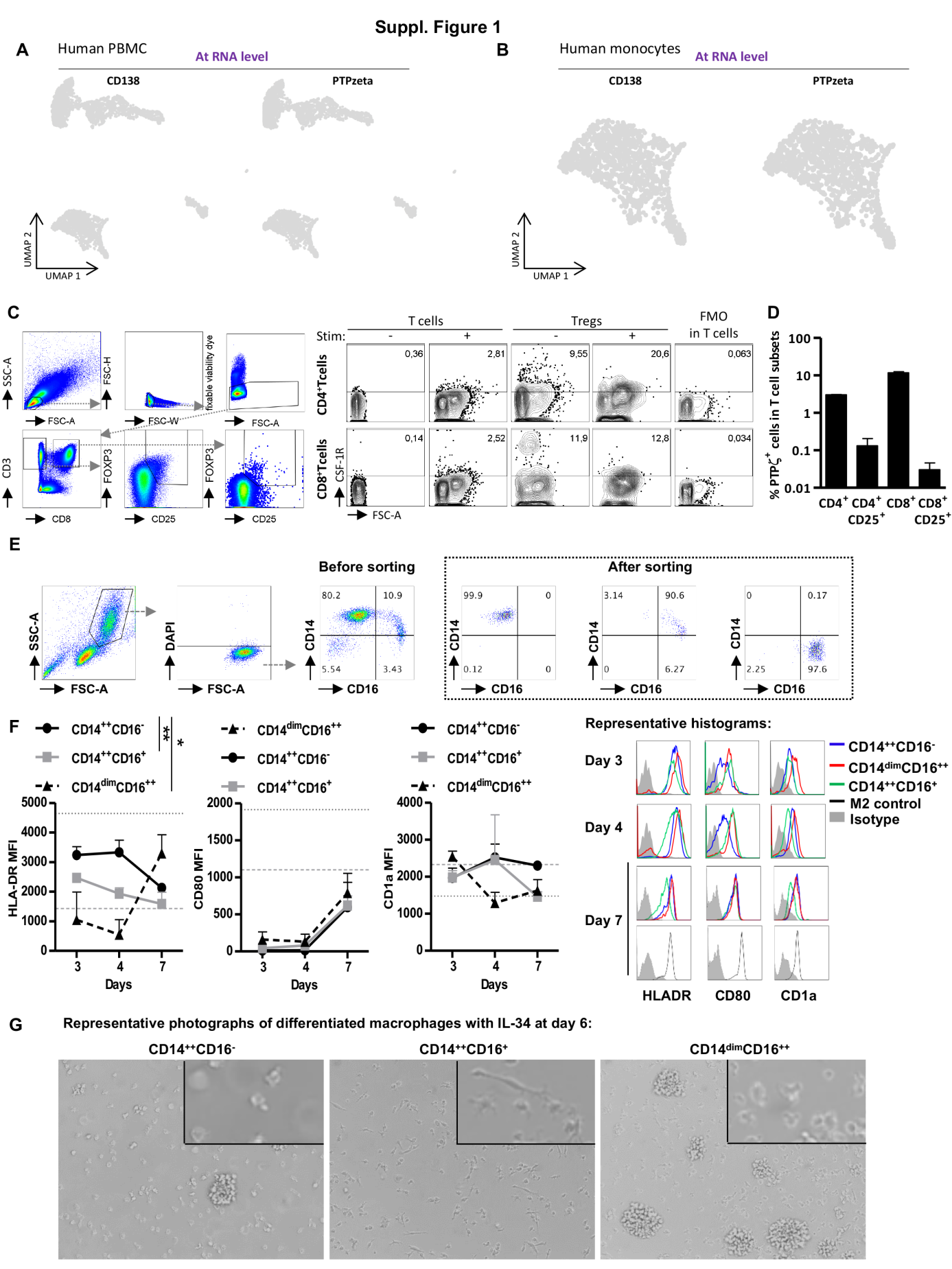
**

**Supplementary figure 1.** **IL-34 acts on monocyte subsets differentially and independently of SDC1 and PTPRZ1 receptors.**

**(A-B)** UMAP visualization of a public dataset of Human PBMCs **(A)** and human monocytes **(B)** single cell RNA-seq from 1 healthy volunteer analyzed for SDC1 and PTPRZ1 expression at transcriptional levels. One point represents one cell. Relative expression level is scaled from grey to dark blue, no expression was detected for both genes. **(C)** Representative gating strategy and staining to identify CD4^+^ (CD8^-^) or CD8^+^ T cells, and CD25^+^FOXP3^+^ Tregs (left) and to analyze CSF-1R expression in stimulated or not stimulated FOXP3^+/-^ CD4^+^ or CD8^+^ T cells (right). **(D)** Frequency of PTPζ expressing cells in CD25^+/-^ CD4^+^ or CD8^+^ T cells. n=3. (**E**) Representative gating strategy of monocyte subset sorting by FACS Aria and purity after sorting. **(F)** Monocyte subsets were cultured for 7 days in presence of IL-34 and analyzed for HLA-DR, CD80 and CD1a markers expression. **Left:** Geometric mean of fluorescence +/- SEM out of 3 experiments is represented over time. M1 (dark grey dotted line) and M2 (light grey dashed line) macrophage mean of fluorescence of 3 individuals after 7-day culture is shown. Mann Whitney U test, *p<0.05, **p<0.01. **Right**: Representative histograms of FACS staining. CD14^++^CD16^-^ (blue line), CD14^dim^CD16^++^ (red line) and CD14^++^CD16^+^ (green line). Isotypic control is shown in filled grey. **(G)** Representative photos of monocyte subsets after 6-day culture with IL-34. The zooms show the presence or absence of dendrites.

**
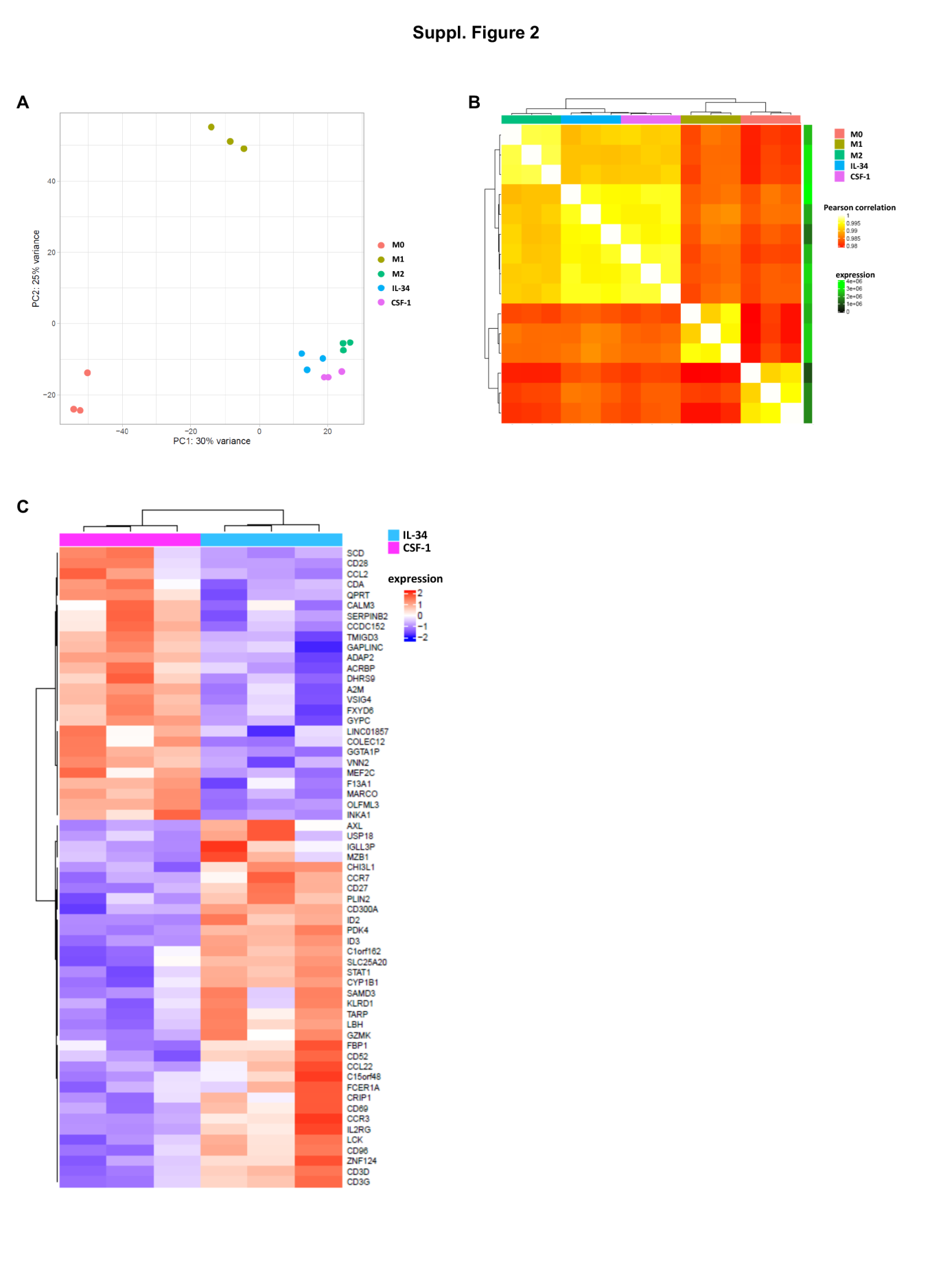
Supplementary figure 2. Transcriptomic bulk RNAseq analysis of macrophages.**

CD14^++^ monocytes were cultured for 6 days with IL-34 or CSF-1 and analyzed by DGEseq for gene expression. **(A)** Principal Component Analysis (PCA) on IL-34-differentiated macrophages (IL-34) in comparison with CSF-1-differentiated macrophages (CSF-1), macrophages type 1 (M1) and type 2 (M2), and monocytes before culture (M0). **(B)** Hierarchical clustering of Pearson correlation coefficient. The colors represent Pearson correlation values: red means positive correlation and blue means negative correlation. Each column represents one sample. The barcolor corresponds to experimental conditions. **(C)** 3′ digital gene expression RNA-sequencing analysis was performed on IL-34- (right) and CSF-1-differentiated (left) macrophages after 6 days of culture. Expression levels of differentially expressed genes are presented as a heatmap; low expression levels are in blue, mean expression levels are in white and high expression levels are in red. Each column represents one sample.

**
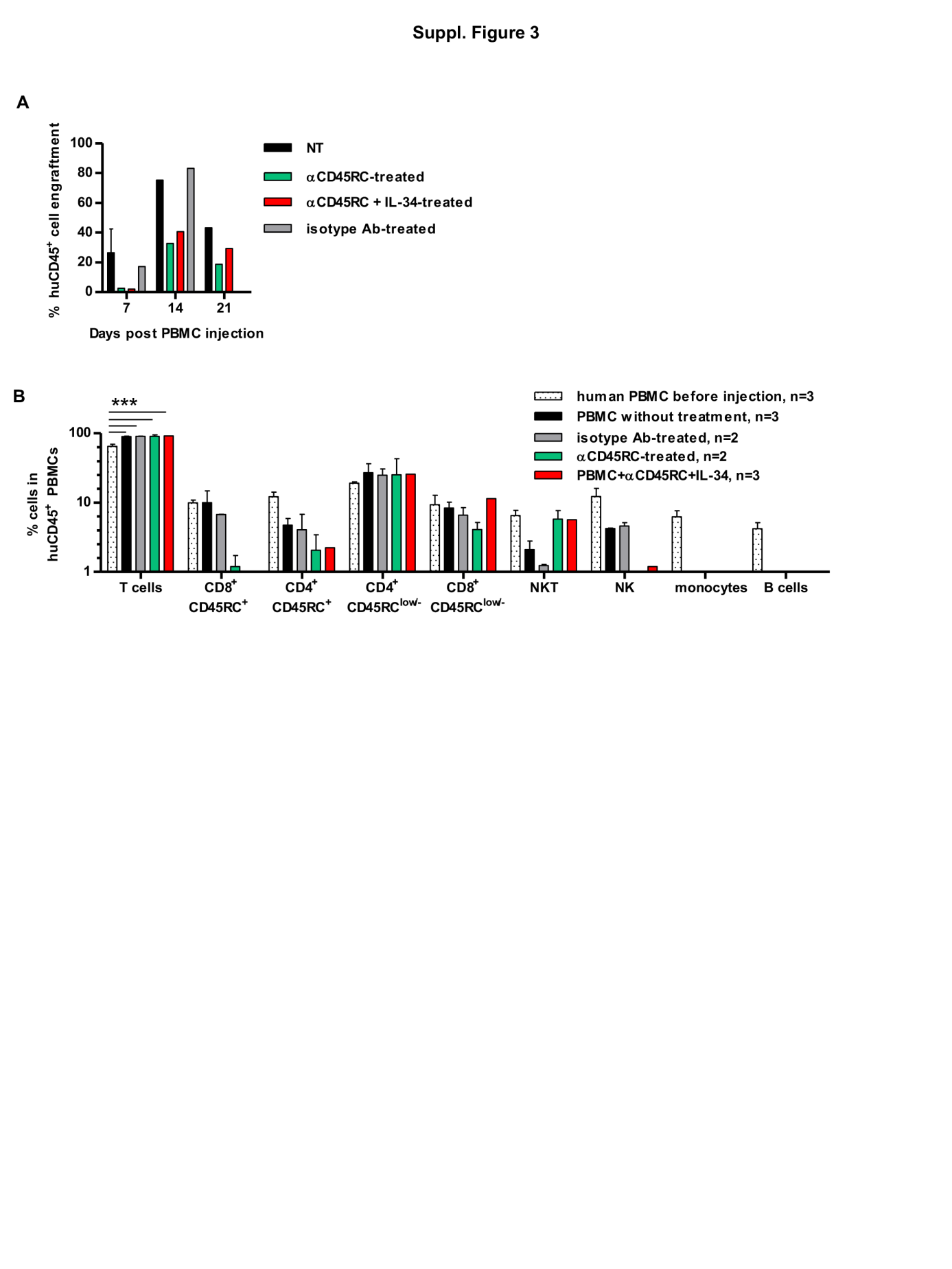
Supplementary figure 3. Follow-up of human cells in NSG mice injected with PBMCs.**

Mice were injected with human PBMCs and depleted or not (NT for not treated in black and isotype control in grey) with anti-CD45RC mAbs (red and green) and treated (red) or not (green) with IL-34 protein. **(A)** Engraftment of human cells (hCD45^+^) was followed in the blood over time (days 7, 14 and 21) in NSG mice. **(B)** The different subpopulations of leukocytes (B, T, Tregs, NK, NKT and monocytes) were analyzed among hCD45^+^ cells at day 7 in the blood of mice treated with anti-CD45RC mAbs (green) or isotype control (grey), or co-treated with anti-CD45RC mAbs and IL-34 (red), and in untreated PBMC-injected mice (black) and compared to human PBMCs before injection (dotted white). n=2-3, Two-way ANOVA and Bonferroni post-test, ***p<0.001.

**
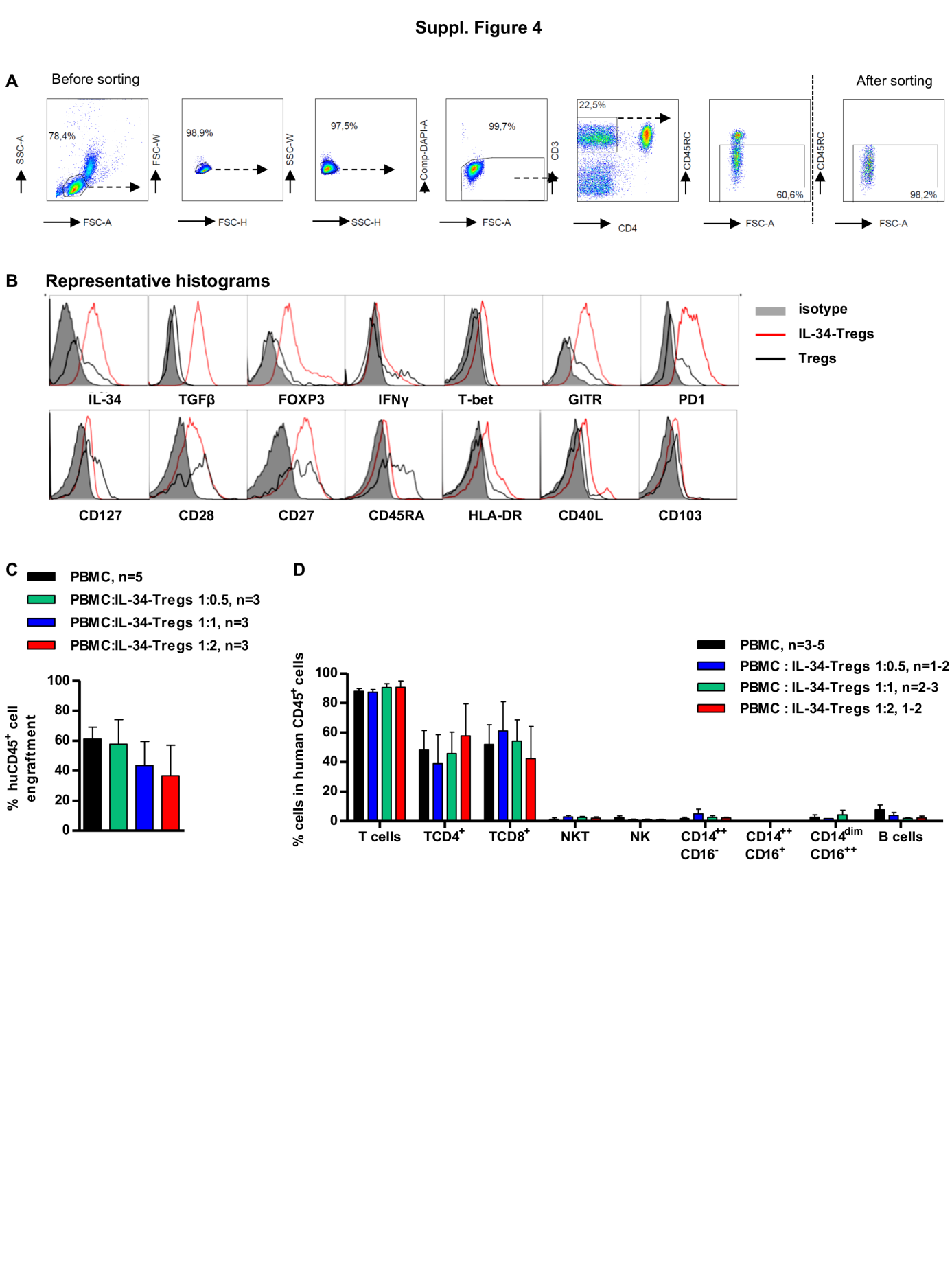
Supplementary figure 4. CD8^+^ Treg cell sorting and persistence after infusion in mice.**

**(A)** Representative gating strategy of FACS Aria sorting of CD3^+^CD8^+^CD45RC^low/-^ Tregs from blood of a healthy volunteer and purity after sorting. **(B)** Representative histograms of FACS staining on IL-34-Tregs (red) vs. fresh Tregs (black) after 14 days of culture. Isotypic control is shown in filled grey. **(C-D)** 15 days after PBMC ± Treg cell infusion, human cells (hCD45^+^) were monitored in the blood of NSG mice **(C)** and the different subpopulations of leukocytes (B, T, Tregs, NK, NKT and monocytes) were analyzed **(D)**.
